# Assessing the optimal location for alcohol-based hand rub dispensers in a patient room in an intensive care unit

**DOI:** 10.1186/1471-2334-13-510

**Published:** 2013-10-31

**Authors:** Matthijs C Boog, Vicki Erasmus, Jitske M de Graaf, Elise (A) HE van Beeck, Marijke Melles, Ed F van Beeck

**Affiliations:** 1 Department of Public Health, Erasmus MC, P.O. Box 2040, 3000, CA, Rotterdam, The Netherlands; 2 Faculty of Industrial Design Engineering, Delft University of Technology, Landbergstraat 15, 2628CE Delft, The Netherlands

**Keywords:** Patient safety, Hand hygiene, Alcohol-based hand rub dispensers

## Abstract

**Background:**

The introduction of alcohol-based hand rub dispensers has had a positive influence on compliance of healthcare workers with the recommended guidelines for hand hygiene. However, establishing the best location for alcohol-based hand rub dispensers remains a problem, and no method is currently available to optimize the location of these devices. In this paper we describe a method to determine the optimal location for alcohol-based hand rub dispensers in patient rooms.

**Methods:**

We composed a method that consists of a combination of qualitative and quantitative research methods. Firstly, different arrangements of dispensers were determined based on the results of two types of assessment: workflow observations and interviews with nurses and physicians. Each arrangement was then evaluated using two types of assessment: interviews with nurses and physicians and electronic measurements of the user frequency of the dispensers. This procedure was applied in a single-bed patient room on a thoracic surgery intensive care unit.

**Results:**

The workflow observations revealed that the activities of patient care were most often at the entrance and near the computer at the right side of the test room. Healthcare workers stated that the location of the dispenser should meet several requirements. Measurements of the frequency of use showed that the dispenser located near the computer, at the back of the room, was used less frequently than the dispenser located near the sink and the dispenser located at the entrance to the room.

**Conclusion:**

The applied method has potential for determining the optimal location for alcohol-based hand rub dispensers in a patient room. Workflow observations and the expressed preferences of healthcare workers guide the choice for the location of alcohol-based hand rub dispensers. These choices may be optimized based on measurement of the frequency of use of the dispensers.

## Background

Although hand hygiene reduces the risk of transmission of infectious agents [1], compliance by healthcare workers (HCW) with the recommended instructions for hand hygiene is low [2]. The introduction of alcohol-based hand rub (ABHR) dispensers is reported to have a positive influence on hand hygiene compliance [3-6], which is attributed to (amongst other factors) the potential for increased access and the visibility of this device.

Greater accessibility has been achieved by placing ABHR dispensers in the vicinity to a bed: e.g. mounted on all patients’ beds [6] or placed at each bedside [5], or by distributing individual bottles of ABHR to HCW [4,6]. Thomas et al. reported that the location of the ABHR dispenser is of more importance than the quantity of ABHR dispensers on a surgical intensive care unit (ICU) [7]. In their study, the ABHR dispensers were secured at the end of a trapeze-bar apparatus which was connected to the patient’s bed. In this way the dispenser remains at eye level for attendants standing at bedside. This location resulted in a higher volume of use for ABHR compared to placing dispensers at the customary locations (e.g. on walls inside/outside the patient rooms, and adjacent to the lavatories) or placing the dispensers at customary locations in greater quantity. Birnbach et al. used a simulation in a real-size replica to evaluate the location of an ABHR dispenser; they found that when the dispenser was in clear view of the physicians this resulted in better compliance [8]. Nevo et al. reported that compliance was improved when the front of the dispenser and an accompanying poster were in the line of sight on entering the room [9].

The optimal location of an ABHR dispenser is associated with its potential for increased access (e.g. close to the point of care, unobstructed access) and visibility. However, due to the variety in the layout of patient rooms in different hospitals, there is no standardized location for ABHR dispensers, and no method is available for establishing the best location for these dispensers.

Therefore, we composed a method to determine the optimal location for an ABHR dispenser in a patient room. This method consists of three types of assessment: workflow observations, interviews with nurses and physicians, and electronic measurements of ABHR dispenser usage. Workflow observations and interviews with HCW were used to identify possible preferred locations for an ABHR dispenser in the test room. These locations were then evaluated with electronic measurements of the frequency of use of the ABHR dispensers and by interviewing HCW.

This paper describes the results of the application of this method on an ICU in a single patient room.

## Methods

### Description of the study method

#### Design

**Figure 1:**
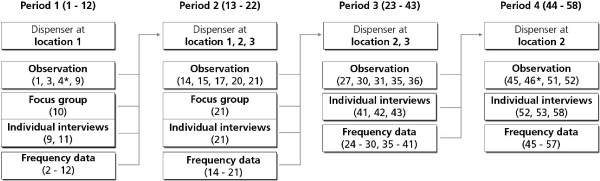
**Workflow observations (‘Observation’), interviews (‘Focus group’ and ‘Individual interviews’) and frequency data of the used dispensers (‘Frequency data’) resulted in new test locations for the ABHR dispensers in a subsequent period.** For each of the four periods, the number of study days is given between brackets. *On these days, two observations were performed.

Different arrangements of dispensers were determined based on the results of workflow observations and interviews with HCW (focus group discussions among nurses, and individual interviews with nurses and physicians). Each arrangement was then evaluated using the results of interviews and electronic measurements of the user frequency of the dispensers. Findings of each evaluation round were used to further optimize the final dispenser arrangement. In this way, four different arrangements of dispensers were evaluated to determine the most optimal location, i.e. combinations of locations 1–3; during test periods 1–4 (see Figure 1).

The study started in January 2011 and lasted 58 days.

#### Materials

During the study a touchless dispenser was used; a built-in electronic sensor registers movements. When the hands are placed within a range of 10 cm under the sensor, a dose of 1.5 ± 0.5 ml ABHR is dispensed. The dispenser contains a 700 ml ABHR refill.

The dispenser dates and time stamps the dispensing act and multiple dispenses directly after each other are registered as one act. This information can be retrieved and transferred when the dispenser is opened and connected to a PC or laptop.

During the study, three electronic ABHR dispensers were used.

#### Procedure

**Figure 2:**
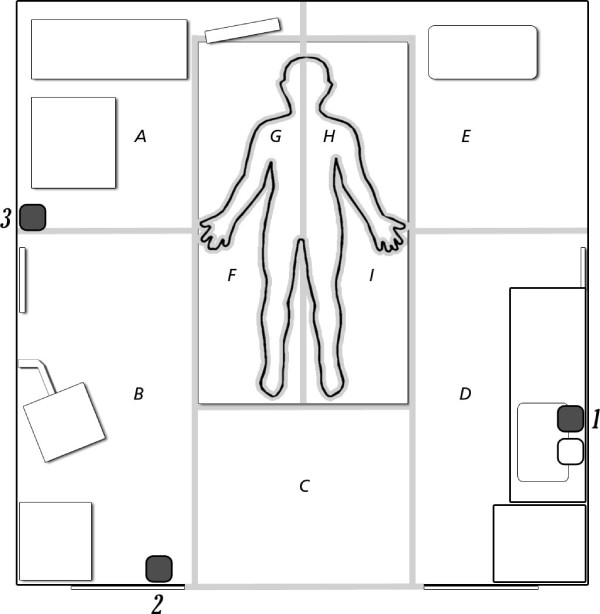
**Patient room layout.** Site **A**: monitor, ventilator, pedestal cupboard, gloves; Site **B**: bin, computer, cabinet; Site **C**: entrance to the test room; Site **D**: sink, cabinet, gloves, alcohol-based hand rub dispenser, soap dispenser, bin, shelf; Site **E**: drip; Site **F**: right side of the bed; Site **G**: right side of the patient; Site **H**: left side of the patient; Site **I**: left side of the bed. ABHR dispenser locations: 1 = location 1; 2 = location 2, 3 = location 3.

During the first period, an ABHR dispenser was placed on the left wall of the test room (from the point of view of the patient), above the sink at the original location (Figure 2, location 1).

The procedure consists of three steps:

– HCW were interviewed about advantages and disadvantages of the location(s) of the ABHR dispenser(s) in the test room. They were also asked about preferred ABHR dispensers locations in the test room. Workflow observations were made to find sites in the test room where HCW were most frequently. In this way, preferred locations for ABHR dispensers were established;

– ABHR dispenser locations were evaluated by interviewing HCW and by measuring the frequency of use of the ABHR dispensers;

– At the start of a new test period, additional ABHR dispensers were placed or ABHR dispensers were removed.

This process was repeated three times (Figure 1). In total, four different arrangements were evaluated during four consecutive test periods.

For example, in the first period the nurses indicated their preferred locations to be on the left wall of the test room and on the right wall of the test room near the computer. Physicians indicated their preferred locations to be on the left wall (near the sink) and at the entrance to the room (near the cabinet). Therefore, based on the results of the interviews and workflow observations, for period 2 we placed additional dispensers at the entrance to the patient room (Figure 2, location 2) and on the wall to the right of the patient near the computer (Figure 2, location 3).

### Setting and participants

The study was conducted in a thoracic surgery ICU that consists of 12 private bedrooms, in a university teaching hospital located in Rotterdam. At this hospital, all new employees are informed about the Dutch guidelines for hand hygiene, based on the five moments described by the WHO [10]. In all patient rooms, an ABHR dispenser is located, just above the sink, next to the soap dispenser. The soap dispenser was remained in use throughout the study period. The entrance to the each room can be closed by means of two manual sliding doors. The present study was conducted in one [test] room only. The layout of the test room is shown in Figure 2. The study did not include the collection of any patient data.

The participants were 8 ICU physicians and 12 ICU nurses. The physicians participated in the individual interviews only, whereas the nurses participated in the focus group discussions and/or in the individual interviews.

All study participants were HCW and no children, parents or guardians were observed. The institutional review board of Erasmus MC Rotterdam provided a waiver for this study. Written informed consent for HCW observations was not requested nor obtained. All participating HCW were made aware that this was a study and that participation was voluntary.

### Data collection

The first period lasted 12 days, the second period 10 days, the third period 21 days and the fourth period lasted 15 days.

#### Observation of the workflow of nurses and physicians

During each of the 4 periods, the workflow of the HCW was observed during 4 or 5 days, for 1 or 2 hours a day (between 8.00 am and 5.00 pm, but at no specific time). The workflow observations were performed by two researchers (MCB and AHEvB). When a nurse and a physician were in the room, the physician was observed. A physician was followed until he/she left the room, or the observation had taken 1 hour. A nurse was followed until he/she left the room, or a physician entered the room, or the observation had taken 1 hour.

The workflow of nurses and physicians was registered by making use of a map (Figure 2). During the observations, the route of the hand(s) of the nurses and physicians was registered by writing down the sites the participating nurses and physicians entered with their hand(s). The observer sat on a chair in site B (Figure 2).

#### Focus group discussions and individual interviews (qualitative research)

The focus group discussions took about 30 min. These group discussions were conducted to obtain a wide range of information on the topic and were led by a moderator (MCB) and supported by an assistant (AHEvB). In periods 1 and 2, 10 and 8 nurses, respectively, participated in the focus group discussions. Individual interviews were conducted with nurses during periods 3 and 4, and with physicians during all 4 periods because their schedules did not allow focus group participation. Eight nurses participated in 12 individual interviews and 8 physicians participated in 8 individual interviews. All interviews took 4–10 min each. An interviewer (AHEvB or MCB) led all interviews.

All focus group discussions and individual interviews were tape recorded and fully transcribed after the end of period 4. During all interviews (focus group discussions and individual interviews) participants were asked about the advantages and disadvantages of the location(s) of the dispenser(s) used in a specific test period, and the locations they would prefer themselves for an ABHR dispenser in the test room.

During periods 3 and 4, instead of organizing focus group discussions six of the participating nurses were individually interviewed.

#### Frequency data of the ABHR dispenser

For each of the 4 periods, the number of dispenses per day for each ABHR dispenser in use was calculated with Microsoft Office Excel 2003 (Microsoft, Redmond, Washington) and Matlab 7.12.0 (R2011a) (MathWorks, Natick, United States).

### Analysis

After each period, the observed frequencies that sites were visited, as well as the results of interview data and electronically measured frequency of use of the dispensers, were collected and discussed by the research team. These outcomes resulted in a new arrangement of the dispensers after the first, second and the third periods.

At the end of the fourth period, aggregate data from the workflow observations, the focus group discussions/interviews and the electronic frequency of dispenser usage were analyzed.

The total frequency distribution that sites were visited by nurses and physicians during the workflow observation was compared using the χ^2^ test for independence. Site F was compared with site I to gain information on how nurses and physicians physically approach the patient’s bed, using the χ^2^ test for goodness-of-fit.

Interviews and focus group discussions were transcribed verbatim. Computer-assisted thematic analysis using NVivo 8 software (QSR International Inc., Doncaster, Australia) was conducted on these transcripts. Using NVivo software the verbatim content of the interviews and focus groups was organized and subsequently analyzed by the researchers (MCB, JMdG, VE).

When two or more dispensers were placed in the test room during a specific period, the total frequency of use was compared between the dispensers. Statistical analysis was performed using the χ^2^ test for goodness-of-fit. The results were considered significant when p < 0.05.

## Results

### Workflow observations

During workflow observations HCW (nurses and physicians combined) visited the back of the test room the least number of times, i.e. site A and site E (Figure 2, Table 1). Site C (near the entrance to the test room) and site B (close to the computer) were the most frequently entered by HCW. Nurses approached the patients more frequently at the patient’s right side: site F compared to site I (p < 0.001) (Table 1); however, this result was not the same for physicians (p = 0.480). The total frequency distribution that sites were visited differed between nurses and physicians (p < 0.001).

**Table 1:** The total frequency distribution that sites were visited by nurses and physicians during the workflow observations

	**Nurses**	**Physicians**	**Nurses and physicians combined**
Site A: monitor, ventilator, pedestal cupboard, gloves	96 (3.6%)	3 (0.9%)	99 (3.3%)
Site B: bin, computer, cabinet	574 (21.7%)	59 (17.6%)	633 (21.3%)
Site C: entrance to the test room	619 (23.4%)	97 (29.0%)	716 (24.0%)
Site D: sink, cabinet, gloves, ABHR dispenser, soap dispenser, bin, shelf	420 (15.9%)	59 (17.6%)	479 (16.1%)
Site E: drip	131 (5.0%)	9 (2.7%)	140 (4.7%)
Site F: right side of the bed	407 (15.4%)*	33 (9.9%)**	440 (14.8%)
Site G: right side of the patient	162 (6.1%)	17 (5.1%)	179 (6.0%)
Site H: left side of the patient	58 (2.2%)	19 (5.7%)	77 (2.6%)
Site I: left side of the bed	176 (6.7%)*	39 (11.6%)**	215 (7.2%)

Differences in the numbers of site visits between the left and right side of the patient (by χ^2^ test for goodness-of-fit):

*: p < 0.001.

**: p = 0.480.

### Interviews about requirements for ABHR dispenser locations

According to the participants, the location of an ABHR dispenser in the test room has to meet several requirements:

#### The dispenser has to be in the line of sight

Participants mentioned that it is important that the ABHR dispenser is in the line of sight (“*You automatically use the dispenser when you see it*” - a physician), especially when entering the room (“*It would be better if you could see the dispenser immediately, but that’s simply not possible because you have a bed there*.” - a nurse). Participants mentioned that location 2 is not visible when entering the room (“*You walk inside and then you don’t look behind you to see if there’s a dispenser hanging there*” - a physician). Some nurses mentioned that the dispenser at location 1 is not visible and the dispenser at location 3 is not noticeable, partly due to the “busy corner”.

#### The dispenser has to be located on the workflow route

Participants suggested that the ABHR dispenser should be located on their route during workflow, preferably near the entrance to the test room. (“*The dispenser at location 2 could be convenient to use when entering or leaving the room. Before you leave the room you use some alcohol*” - a physician). Nurses mentioned that location 2 is on their walking route; especially when they enter or leave the room, walk to the computer or to the patient, or walk between the computer and the patient. (“*I like location 2, at the exit. It’s close to the computer, so you’re able to use it easily. Otherwise, you would have to make a detour [to use the dispenser at location 1]”* - a nurse). Some nurses mentioned that location 2 is a central location which is easy to approach from both the left and right side of the test room, in contrast to locations 1 and 3 which are located at opposite sides of the room. In addition, some participants mentioned that an ABHR dispenser placed on their walking route reminds them to use an alcohol-based hand rub (“*I would appreciate something that reminds me to disinfect my hands when entering or leaving the room*.” - a nurse).

#### The dispenser should be located in the proximity of certain objects and should be within reach during certain procedures

Participants mentioned that the ABHR dispenser should be located in the proximity of certain objects and should be within reach during certain procedures. Some participants mentioned that the ABHR dispenser has to be located near the sink (at location 1) because some use soap and water as well as alcohol-based hand rub (“*Before an invasive procedure, you wash your hands with soap and water, then you put on your coat and then you rub your hands with alcohol. In that case it’s useful when the dispenser is placed near the sink*” - a physician). On the other hand, some participants mentioned that they find it convenient to have an ABHR dispenser within reach (location 3) while giving medical care to a patient or examining a patient. (“*When we examine a patient, we stand on the right-hand side of the patient. That’s how we’re trained. When standing there, it’s easy to turn around, use the dispenser, examine the patient, turn around again and use the dispenser again afterwards*.” - a physician). Nurses mentioned that the ABHR dispenser should be close by during specific procedures (“*I often use this dispenser because at this side of the room you are busy with many things like urine, blood, drips and medication. You’re busy with a lot of things on that side*” - a nurse), or near the computer (“*We often walk straight to the computer after entering the room. Because of that, I think it’s easier to use a dispenser that is placed close to the computer than a dispenser that is placed close to the sink*.” - a nurse). It was also mentioned that the location of gloves, tissues and the bin near location 1 is convenient.

#### People or objects should not obstruct the route to the dispenser

Participants mentioned that the route to the ABHR dispenser should not be obstructed by people or objects. (“*There should be no obstacle between you and the dispenser. If you have to walk around an obstacle, you’re less likely to use the dispenser*” - a nurse). According to some physicians the route to location 2 is less frequently obstructed by nurses. Some physicians mentioned that the pedestal cupboard or the mechanical ventilator sometimes obstructs the route to location 3.

#### The dispenser should be located at a familiar location

Prior to this research, there was already a dispenser located above the sink (location 1) in each patient room. Some participants mentioned that the dispenser should be located at this familiar location. (“*Because I’m used to this location [i.e. location 1], I think it’s the most practical location for me”* - a physician).

### Frequency data dispensers

During the first period, the ABHR dispenser at location 1 was used 336 times. During the second period, the ABHR dispenser at location 1 was used 152 times, the dispenser at location 2 was used 131 times and the ABHR dispenser at location 3 was used 23 times. During the third period, the ABHR dispenser at location 2 was used 358 times and the ABHR dispenser at location 3 was used 86 times. During the fourth period, the ABHR dispenser at location 2 was used 329 times.

**Figure 3:**
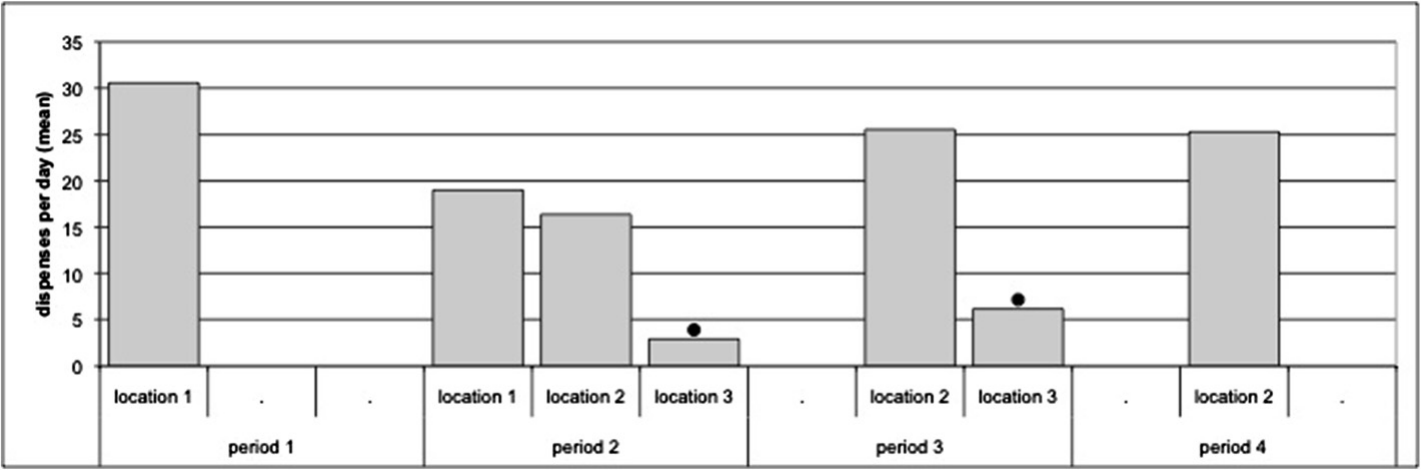
**User frequency of the ABHR dispensers during the four periods.** During period 2 the dispensers at location 1 and location 2 were used more frequently than the dispenser at location 3 (p < 0.001). During period 3 the dispenser at location 2 was used more frequently than the dispenser at location 3 (p < 0.001). ● Total dispenses per day differed significantly from other dispensers in use during same period (p < 0.001).

During the second period, the dispenser at location 3 (near the computer at the right side of the patient) was used less frequently than the dispenser at the entrance (location 2) and the dispenser at the sink (location 1) (p < 0.001) (Figure 3). During the third period the dispenser near the computer (at the right side of the patient) was used less frequently than the dispenser at the entrance (p < 0.001).

When the results of the workflow observations, interviews and frequency of use of the dispensers are combined, it seems that location 1 (near the sink) and location 2 (at the entrance) are both preferred locations for an ABHR dispenser in the test room.

## Discussion

For the present study, we applied a mixed method to find the optimal location for an ABHR dispenser in a single patient room on an ICU.

The evaluated locations were selected based on the combined results of workflow observations and interviews (location 2 near the room entrance, and location 3 near the computer) or based on the results of interviews alone (location 1 near the sink). The results of the workflow observations did not indicate that the area near the sink is the preferred area for an ABHR dispenser. However, the frequency of use of the dispensers revealed that the dispenser near the sink and the dispenser at the entrance to the test room were used more frequently compared to the dispenser near the computer. Although workflow observations and interviews are useful means to find possible preferred locations, they do not seem to be conclusive. The additional measurement of the frequency of dispenser usage has added value in the evaluation of preferred dispenser locations.

The electronic measurements of the user frequency of the ABHR dispensers revealed that the dispenser at the entrance to the test room (location 2) was often used. It seems that the reasons for this frequent use are its location on the workflow route of HCW and the unobstructed access to the dispenser.

The user frequency of the ABHR dispensers further revealed that the dispenser near the computer (location 3) was used less frequently than the dispensers near the sink (location 1) and at the room entrance (location 2). The dispenser at location 3 was probably not sufficiently noticeable due to the presence of other objects in this part of the room. During period 1 the nurses indicated a preferred location on the right wall of the test room; however, due to the placement of the computer at this often-visited site (Figure 2, site B), the dispenser could not be placed at this location. Therefore, the dispenser was placed on the right wall, but slightly further into the test room (site A). Workflow observations showed that site A was the least visited site in the room, which may be the reason of the less frequent use of the dispenser. In addition, after this test period, the participants of the interviews and a focus group discussion mentioned that various persons might more often obstruct the access to this particular ABHR dispenser.

Electronic measurements of the user frequency of the ABHR dispensers revealed that the ABHR dispenser near the sink (location 1) was most often used in the test room. The familiarity of this location (mentioned by participants during focus group discussions and individual interviews) seems an advantage of this location compared to the two other locations. For example, Erasmus reported that the hand hygiene behavior of physicians and nurses is strongly influenced by habit [11]. The frequent use of the dispenser at location 1 near the sink could be due to such habitual behaviour. However, it should be noted that a high frequency of use is not necessarily related to a high level of compliance with hand hygiene guidelines.

For the present study, our methodology combines objective measures (workflow observations, frequency of dispenser use) and subjective measures (user experience) to determine the optimal location for an ABHR dispenser and might be applied to all patient rooms. Although the outcomes from this study cannot simply be generalized to other types of patient rooms, hospital units or hospitals, the methodology can be applied in different settings (e.g. other wards and/or hospitals). This will enable hospital managers and infection control staff to identify the optimal locations in their own specific situation. The applied method has some limitations and needs further refinement.

We used electronic frequency measurements to evaluate the dispenser locations and not compliance with the recommended guidelines for hand hygiene (which is a more superior indicator for the preferred location of an ABHR dispenser). According to the WHO guidelines on hand hygiene in health care, hand hygiene should be applied before touching a patient, before a clean/aseptic procedure, after body fluid exposure risk, after touching a patient and after touching patient surroundings [10]. An ABHR dispenser should be located where HCW are enabled to use this device at these moments. We assumed that when a dispenser is used more frequently, the dispenser was placed at a better location. However, compliance and frequency of use are not necessarily highly correlated. Bischoff et al. found that with the introduction of an alcohol-based waterless antiseptic solution, the total counts of soap, chlorhexidine and alcohol-based waterless antiseptic solution did not increase despite a directly observed increase in hand hygiene compliance [3].

However, because of our focus group discussions and individual interviews, HCW were aware of the study, which made an unobtrusive observation on compliance with the recommended guidelines for hand hygiene impossible. Under these circumstances, electronic frequency measurements probably better reflect actual HCW behavior than observation data. However, it should be noted that this behavior does not equate to compliance with the WHO guidelines for hand hygiene.

Another limitation of this study is the short duration of the periods that the dispensers were placed at the new locations. The length of each period may have been insufficient for physicians and nurses to get used to the new locations. In addition, the location above the sink (location 1) is the standard location for an ABHR dispenser in a patient room at this ICU. Based on the frequency of use of the ABHR dispensers, this familiar location 1 would seem most favorite after period 2. It seems that the rationale behind this score was generally based on habit, e.g. “You know the dispenser is at your right, so you walk to the right” (a physician during an interview). But during the same interview this physician indicated that an ABHR dispenser should preferably be located closer to the door of the test room. In order to investigate the optimal location HCW need time to get used to new and unusual locations (2 & 3). In addition, because HCW were accustomed to location 1, this may have influenced the frequency with which the dispenser was used at this location compared to the others. Therefore, it is difficult to compare location 1 with locations 2 and 3, and to declare a ‘winner’ in the test room.

We did not collect any data on the patients that were in the test room during the study, even though the condition of the individual patients might have influenced the use of ABHR dispensers at a particular location. To minimize this effect, the study was conducted at a ward with a homogeneous patient population. All patients at this ward underwent thoracic surgery followed by a short stay (12–24 h) in the ICU for the vast majority of patients. In addition, only frequency data between ABHR dispensers within a specific period were compared.

Workflow observations were used to find sites that are frequently visited by nurses and physicians. We assumed that the frequency of use of the dispensers is influenced by the number of times the nurses and physicians are in the proximity of the dispenser during workflow. Hence, a dispenser should be placed at an often-visited site. However, it is unclear whether this link actually exists. In addition, ABHR dispensers should probably be placed at locations where they enable HCW to use ABHR when necessary. Therefore, workflow observations preferably need to focus on the need for ABHR dispensers during the tasks of HCW, rather than only establishing the most frequently visited sites. Therefore, the present study should be seen as a first step towards a mixed methods approach to assess the best locations for a dispenser. In subsequent steps, the development and application of workflow observation methods related to tasks and opportunities for hand hygiene could certainly improve this approach.

To our knowledge, this is the first study to use a mixed method to find a preferred location for an ABHR dispenser in a patient room. Birnbach et al. used a methodology which utilizes simulation-based testing in a real-size replica of a hospital room [8]. An ABHR dispenser that was in direct view of physicians as they observed the patient was compared to a location that was hidden when facing the patient; they found that more physicians used the ABHR dispenser before patient examination when the dispenser was in direct view [8]. Nevo et al. also compared ABHR dispenser locations and found that placing the ABHR dispenser in the line of sight on entering the room, instead of a less conspicuous location, improved hand hygiene compliance [9]. Thomas et al. found that a conspicuous display and immediate proximity to patients of ABHR dispensers resulted in a significant increase in daily consumption of ABHR [7]. The dispensers were secured at the end of a trapeze-bar apparatus which was connected to the patient’s bed, in plain view of HCW, instead of at the wall, or inside/outside the patient rooms or adjacent to lavatories [7]. The method we applied also compares dispenser locations, but the test locations are a combined result of focus group discussions, individual interviews and workflow observations. A preferred location is found by comparing these locations during an iterative process.

## Conclusions

The applied mixed method has potential for determining the preferred locations for an ABHR dispenser in a patient room. Using this method a location can be determined by taking into account several requirements for a preferred location, and not just one single requirement, such as the visibility of the ABHR dispenser. Workflow observations and expressed preferences of HCW can guide the choice of locations for the ABHR dispensers. These choices may be optimized based on measurement of the frequency of dispenser usage.

## Competing interests

The authors declare that they have no competing interests.

## Authors’ contributions

Study design: MCB, VE, AHEvB, MM, EFvB. Data collection: MCB, AHEvB. Data analyses: MCB, VE, JMdG, AHEvB, MM, EFvB. All authors contributed to the drafting of the manuscript and approved the final manuscript.

## Pre-publication history

The pre-publication history for this paper can be accessed here:

http://www.biomedcentral.com/1471-2334/13/510/prepub
